# *In Vivo* acrylamide exposure may cause severe toxicity to mouse oocytes through its metabolite glycidamide

**DOI:** 10.1371/journal.pone.0172026

**Published:** 2017-02-09

**Authors:** Duru Aras, Zeynep Cakar, Sinan Ozkavukcu, Alp Can, Ozgur Cinar

**Affiliations:** 1 Laboratories for Stem Cells and Reproductive Biology, Department of Histology and Embryology, Ankara University School of Medicine, Sihhiye, Ankara, Turkey; 2 Centre for Assisted Reproduction, Department of Obstetrics and Gynecology, Ankara University School of Medicine, Cebeci, Ankara, Turkey; Peking University Third Hospital, CHINA

## Abstract

High acrylamide (ACR) content in heat-processed carbohydrate-rich foods, as well as roasted products such as coffee, almonds etc., has been found to be as a risk factor for carcinogenicity and genotoxicity by The World Health Organization. Glycidamide (GLY), the epoxide metabolite of ACR, is processed by the cytochrome P-450 enzyme system and has also been found to be a genotoxic agent. The aim of this study was to determine whether ACR and/or GLY have any detrimental effect on the meiotic cell division of oocytes. For this purpose, germinal vesicle-stage mouse oocytes were treated with 0, 100, 500, or 1000 μM ACR or 0, 25, or 250 μM GLY *in vitro*. *In vivo* experiments were performed after an intraperitoneal injection of 25 mg/kg/day ACR of female BALB/c mice for 7 days. The majority of *in vitro* ACR-treated oocytes reached the metaphase-II stage following 18 hours of incubation, which was not significantly different from the control group. Maturation of the oocytes derived from *in vivo* ACR-treated mice was impaired significantly. Oocytes, reaching the M-II stage in the *in vivo* ACR-treated group, were characterized by a decrease in meiotic spindle mass and an increase in chromosomal disruption. *In vitro* GLY treatment resulted in the degeneration of all oocytes, indicating that ACR toxicity on female germ cells may occur through its metabolite, GLY. Thus, ACR exposure must be considered, together with its metabolite GLY, when female fertility is concerned.

## Introduction

Acrylamide (ACR) is a colorless and odorless industrial compound formed by the hydration of acrylonitrile. It is widely used in the treatment of wastewater, paper/pulp manufacturing, mining, scientific research, surface coatings, textile and cosmetics [1, 2]. Environmental exposure to ACR may happen either by polymer construction or polymer use [3]. Once taken into the body, approximately 50% of ACR is metabolized to its epoxide metabolite glycidamide (GLY) by the cytochrome P-450 enzyme CYP2E1 [4]. Following ACR exposure, ACR is metabolized to GLY, and both molecules can be found in several tissues in the body [5].

ACR was first defined as a neurotoxic agent in 1989, after a detailed study on workers who were exposed to ACR and acrylonitrile for two or more years [6]. It was reported that the levels of hemoglobin adducts, which are biomarkers for ACR exposure [7], were correlated with the degree of peripheral neuropathy [6]. In 2001, Hagmar *et al*. [7] discovered that the reaction products of ACR may also be found in human blood without a known exposure history. Several hypotheses were proposed considering the unknown source of ACR exposure [8–10]. In April 2002, Tareke *et al*. [11] published a comparative analysis that documented the levels of ACR in heat-processed protein- and carbohydrate-rich foods, at 120°C or above, for the first time. This study showed that heated protein-rich foods possess 5–50 μg/kg ACR, whereas ACR content in heated carbohydrate-rich foods such as in potato, beetroot and crisp bread is significantly higher (150–4000 μg/kg). It was also reported that processed foods, such as roasted almonds, coated peanuts, fried potato, biscuits, and coffee, contain high levels of ACR [10]. In June 2002, The United Nations Food and Agricultural Organization (FAO) and The World Health Organization (WHO) held a consultation on “Health Implications of Acrylamide in Food” [12]. The report from the joint FAO/WHO consultation emphasized that ACR consuming diets may lead to severe carcinogenic implications and genotoxicity. Moreover, Hsu *et al*. [13] documented a heat-dependent increase of ACR levels in French fries, fried oil and vapor and noted that “fast-food restaurant workers are potentially subject to occupational hazards from acrylamide inhalation” in their very recently published article. Mojska *et al*. [14] also determined the ACR level in cigarette smoke with liquid chromatography–mass spectrometry in a Polish population and noted that tobacco smoke includes significant amounts of ACR with a total ACR exposure rate for smokers over 50% greater than for non-smokers.

The primary pathway responsible for the ACR genotoxicity is the production of GLY through CYP2E1 [15]. As reviewed by Favor and Shelby in 2005 [16], exposure of spermatozoa or spermatids to ACR or GLY increases the frequency of mutational events. In another study, a stronger effect was observed for GLY than ACR on the male reproductive system, especially on sperm cell viability [17]. In 1986, Sakamoto and Hashimoto [18] reported a decrease in fertility rates and litter size in mice exposed to 1.25 to 24 mg/kg/day ACR for 4 weeks. Sperm counts decreased and an increase in abnormal sperm was observed [18]. Male rats exposed to 4.2 to 7.9 mg/kg/day ACR for 10 weeks were characterized with reduced mounting activities and decreased numbers of sperm deposited in the uterus [19, 20]. It was also shown that 10 weeks exposure of ACR causes testicular atrophy, a decreased testes weight and the degeneration of seminiferous epithelium [21].

One possible mechanism of ACR-related neurotoxicity is the interference of GLY with kinesin-related motor proteins, which also play a crucial role in meiotic division [22]. Both meiotic division and chromosome integrity are fundamental to oocyte maturation, which occurs through a series of events including germinal vesicle (GV) breakdown and extrusion of the polar body. Mature oocytes, undergoing nuclear and cytoplasmic changes from pro-metaphase-I to metaphase-II (M-II), are key to fertilization and embryo development [23]. However, the number of ACR studies focusing on the female reproductive system has been limited. To our knowledge, this study is the first to evaluate the effects of GLY treatment on mouse oocytes. Therefore, the aim of this study was to examine the effect of ACR and its metabolite GLY on mouse oocytes *in vitro* and *in vivo*, focusing on meiotic maturation.

## Materials and methods

All reagents were purchased from Sigma-Aldrich (St. Louis, MO, USA) unless otherwise stated.

This study was carried out in strict accordance with the recommendations in the Guide for the Care and Use of Laboratory Animals of Republic of Turkey Ministry of Food, Agriculture and Livestock. Ethical approval of the study was obtained from Ankara University Local Ethical Board of Animal Experiments (approval no. 2012-17-109). All animals were housed in an appropriate temperature and dark-light cycles controlled room in the Laboratory Animal Unit of Ankara University. Mice were sacrificed by cervical dislocation.

All statistical analyses were performed through an SPSS software package (version 15.0; SPSS Inc., Chicago, IL, USA). A chi-square test was utilized to compare groups. The significance level was set at p < 0.05.

### Isolation of mouse oocytes

The ovarian follicular development of 21-day-old female BALB/c mice (n = 37) was stimulated by the intraperitoneal injection of 0.5 IU pregnant mare’s serum gonadotropin. Animals were sacrificed 48 hours following injection, and oocytes were collected from the ovaries by follicular puncture. Denuded GV-stage oocytes (n = 1084) were transferred into gamete maturation medium (G-IVF Plus, Vitrolife, Sweden) and incubated in a humidified atmosphere of 5% CO_2_ at 37°C for 18 hours.

### ACR and GLY experiments

ACR (purity 99.9%, MW 71.08 g/mol) was dissolved in ultrapure water to prepare a 10 mM stock solution. Working solutions were freshly prepared and dissolved either in a culture medium or 0.9% physiological saline depending on the experimental procedure. The *in vitro* and *in vivo* effects of ACR on oocyte maturation were tested by two different sets of experiments. To test the *in vitro* effects of ACR on oocyte meiotic maturation, GV-stage oocytes (n = 452) collected from female BALB/c mice (n = 15) were cultured in gamete maturation medium containing progressive doses of ACR (0, 100, 500 and 1000 μM) for 18 hours. M-II stage oocytes were then fixed with a microtubule-stabilizing buffer containing 2% formaldehyde and 0.1% Triton-X for 30 minutes at 37°C to assess the meiotic spindle and chromosome morphology.

The *in vivo* effects of ACR were tested by the intraperitoneal injection of ACR to female BALB/c mice (n = 12) for 7 days (25 mg/kg/day). Oocytes were then collected from the ovaries as described above. The maturation stage of the oocytes (n = 342) was determined with an inverted microscope (Olympus, Melville, NY, USA) equipped with a heating stage. M-II stage oocytes were fixed as described above.

To determine whether the *in vivo* effect of ACR was related to GLY metabolism or not, GV-stage oocytes from female BALB/c mice (n = 10) were exposed to 0, 25 or 250 μM GLY *in vitro*. One millimolar stock GLY (purity ≥96.0%, MW 87.08 g/mol) solution was prepared by dissolving 87.08 mg GLY in 1 mL ultrapure water. Working solutions were freshly prepared and dissolved in a culture medium. The maturation of oocytes (n = 290) was monitored for 18 hours and the maturation stage of each oocyte was determined.

### Labeling of oocytes and image acquisition

Fixed oocytes were incubated in a 1:100 dilution of anti-α-tubulin/anti-β-tubulin (1:1 mixture) mouse monoclonal antibodies for 90 minutes at 37°C for the evaluation of meiotic spindle integrity. Oocytes were then washed in phosphate-buffered saline (PBS) and incubated in a 1:100 dilution of an affinity-purified fluorescein isothiocyanate (FITC)-conjugated goat anti-mouse IgG (Jackson ImmunoResearch, PA, USA) for 60 minutes at 37°C. Oocytes were stained with 10 μM 7-aminoactinomycine-D (7-AAD) as a nuclear stain. Finally, oocytes were mounted between glass slides and coverslips with a glycerol/PBS solution (1:1 mixture) containing 25 mg/mL sodium azide as an anti-fading reagent.

Slides were assessed with a laser scanning confocal microscope (Zeiss LSM-510, Jena, Germany) equipped with 488 nm Argon, 543 nm He-Ne and 633 nm He-Ne lasers and a 63× Zeiss Plan-Apo objective. Single and z-axis optical sections were collected, and 3D images were constructed using LSM-510 software.

## Results

### *In vitro e*ffect of ACR on mouse oocytes

**Fig 1:**
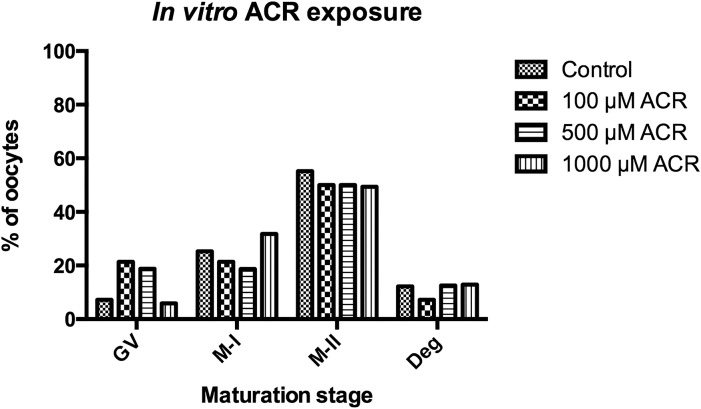
*In vitro* Effect of ACR on Oocyte Maturation. *In vitro* maturation stages of 0, 100, 500 and 1000 μM ACR-treated oocytes following 18 hours of incubation. There was no significant difference between control and ACR-treated groups in terms of oocytes reaching the M-II stage or degenerated oocytes.

**Fig 2:**
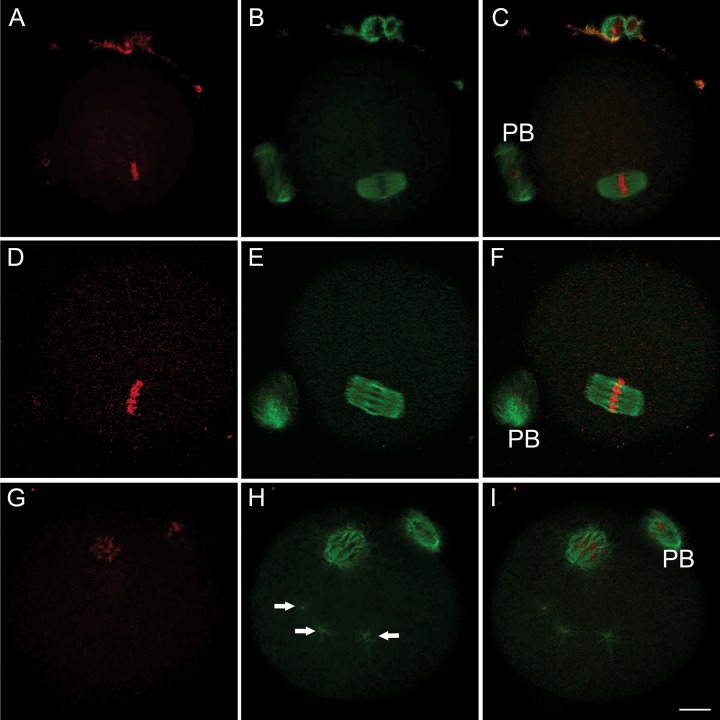
*In vitro and In vivo* Effects of ACR on Meiotic Spindle. *In vitro*-matured M-II stage oocytes in control (**A**-**C**), *in vitro* ACR-treated (**D**-**F**) and *in vivo* ACR-treated (25 mg/kg) groups (**G**-**I**). Representative images in **D-F** were taken from a 1000 μM ACR-treated group. *In vitro* ACR-treated M-II stage oocytes showed no disruption in the meiotic spindle **(green signal)** and chromosome organization **(red signal)** appeared to be intact. *In vivo* ACR-treatment caused a decrease in spindle microtubule mass and disruption in the chromosome alignment. Multiple microtubule-organizing centers (MTOCs) were detected (arrows). PB: polar body. Scale bar: 10 μm.

Meiotic spindle formation during *in vitro* maturation was monitored following treatment to GV-stage oocytes with 100, 500 and 1000 μM ACR. Following 18 hours of incubation, 55.2% of the oocytes reached the M-II stage in the control group. There was no significant difference between the control and ACR-treated groups in terms of meiotic resumption of the oocytes (50.0% for 100 μM, p = 0.613; 50.0% for 500 μM, p = 0.705; and 49.4% for 1000 μM, p = 0.373) (Fig 1). The meiotic spindle was observed in its typical barrel-shape and chromosomes were aligned properly along the metaphase plate in both the control (Fig 2A, 2B and 2C) and ACR-treated (Fig 2D, 2E and 2F) groups. The number of degenerated oocytes was also comparable between the control and ACR-treated groups following 18 hours of incubation (Fig 1).

### *In vivo* effect of ACR on mouse oocytes

**Fig 3:**
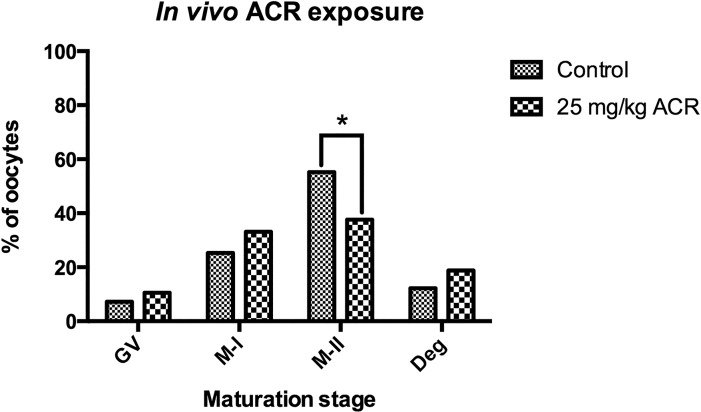
*In vivo* Effect of ACR on Oocyte Maturation. Maturation stages of oocytes isolated from control and ACR-treated mice were presented. *The percentage of M-II stage oocytes was significantly higher in the control group compared to the ACR-treated group (p = 0.001).

Isolated GV-stage oocytes from the ovaries of control or ACR-treated mice were incubated in a maturation medium for 18 hours. Oocytes were then subjected to meiotic resumption analyses. The percentage of M-II stage oocytes in the control group (55.2%) was significantly higher than in the ACR-treated group (37.6%, p = 0.001) (Fig 3). Although the degeneration rate was higher in the ACR-treated group (18.8%) compared to the controls (12.2%), it did not reach statistically significant levels (Fig 3). The majority of the M-II stage oocytes showed a distinct pattern in which the meiotic spindle mass was reduced and the chromosome alignment was disrupted in the ACR-treated group (Fig 2G, 2H and 2I). Moreover, multiple microtubule-organizing centers (MTOCs) were detected in the ACR-treated group (Fig 2H, arrows).

### *In vitro* effect of GLY on mouse oocytes

**Fig 4:**
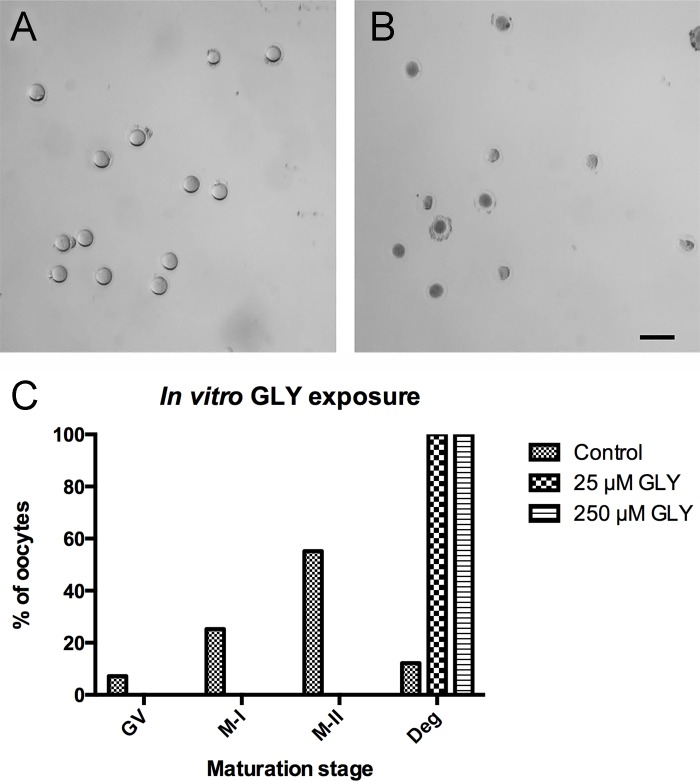
*In vitro* Effect of GLY on Oocyte Maturation. *In vitro*-maturated oocytes were presented in control (**A**) and *in vitro* GLY-treated (**B**) groups. Bar graphs show the *in vitro* maturation stages of 0, 25 and 250 μM ACR-treated oocytes following 18 hours of incubation (**C**). The representative image in **B** was taken from a 25 μM GLY-treated group. The majority of the GV-stage oocytes reached the M-II stage in the control group, whereas all GLY-treated oocytes were degenerated following 18 hours of incubation **(A-C)**. Scale bar: 200 μm.

The discrepancy between the results of *in vivo* and *in vitro* ACR experiments led us to focus on the effects of GLY, the epoxide metabolite of ACR. To this end, isolated GV-stage oocytes were treated with 25 or 250 μM GLY for 18 hours *in vitro*. All oocytes were degenerated in both GLY treated groups, whereas 55.2% of the oocytes reached the M-II stage in the control group (Fig 4).

## Discussion

ACR is an industrial chemical produced in large quantities. Although exposure to industrial forms of ACR is limited to particular occupational groups, the discovery of the high ACR content in heat-processed and/or carbohydrate-rich foods has drawn worldwide attention. ACR has been declared as a priority existing chemical by numerous governments and organizations such as the United Kingdom, Australia, the WHO/FAO Joint Commission, the European Union and the Organization of Economic Cooperation and Development (OECD) [3].

According to our previous experience, particular environmental agents such as dicoumarol [24, 25] and bisphenol-A [24, 25] may affect germline and somatic cells in the female reproductive system. Therefore, we took the potential detrimental effects of ACR and GLY on reproductive health into consideration. To this end, firstly, the *in vitro* maturation of GV-stage oocytes was monitored following *in vitro* ACR treatment. Our findings showed that 100, 500 or 1000 μM ACR exposure did not block the *in vitro* maturation of mouse oocytes. In contrast, a recent study revealed that even low doses (10 and 20 μM) of *in vitro* ACR exposure to cumulus–oocyte complexes may cause a significant decrease in the number of mature oocytes. In the same study, chromosome misalignment and abnormal spindle configurations were distinguished following *in vitro* ACR exposure [26]. On the contrary, we have shown that *in vitro* ACR treatment had no adverse effects on meiotic spindle or chromosomes. Since Liu et al tested the *in vitro* effects of ACR on cumulus-enclosed oocytes, we wanted to examine the effects of ACR on denuded oocytes. As discussed later in this section, we hypothesize that the incubation of oocytes with the granulosa cells may have caused this effect.

Secondly, oocytes derived from *in vivo* ACR-treated (25 mg/kg/day) BALB/c mice were subjected to meiotic resumption analysis. Our findings demonstrated that *in vivo* ACR treatment impaired oocyte maturation. The number of M-II stage oocytes was significantly higher in the control group compared to the ACR-treated groups. Moreover, a diminished meiotic spindle mass and disrupted chromosome alignment were observed in M-II stage oocytes following *in vivo* ACR exposure. Likewise, Duan *et al*. [27] noted that oral ACR administration (10 mg/kg/day and 50 mg/kg/day for 6 weeks) reduced the GV-breakdown and polar body extrusion rates and induced abnormal meiotic spindle rates, as well as reactive oxygen species (ROS) levels and Annexin-V immunopositivity [27]. In the same study, it was reported that litter sizes were significantly smaller following ACR-feeding compared to control mice. In a study by Hulas-Stasiak *et al*. [28], the ovaries of 3 mg/kg/day ACR-treated pregnant guinea pig pups were analyzed. It was demonstrated that *in vivo* ACR exposure caused a decrease in the number of healthy follicles and an increase in the apoptotic cells. Hulas-Stasiak *et al*. also argued that ACR exposure breaks oocyte–cumulus cell connections by disrupting vimentin filaments resulting in apoptosis [28]. Similarly, Wei *et al*. [29] reported that oral ACR exposure causes a significant reduction in body weights, organ weights and the number of corpora lutea in mice, possibly acting through the nitric oxide synthase (NOS)-signaling pathway. In a very recently published study, Dobrovolsky *et al*. [30] evaluated the genotoxicity of ACR by treating rats with similar doses (20 mg/kg/day) of ACR and noted that ACR exposure resulted in DNA damage in the liver but not in the bone marrow, indicating a tissue-specific genotoxic effect of ACR.

Inconsistency between the results of *in vivo* and *in vitro* ACR experiments in our study led us to focus on the effects of GLY, the epoxide metabolite of ACR, which was previously suspected to cause genotoxicity [15, 31]. Sickles *et al*. [22] suggested that ACR displays its toxic effects on peripheral neuropathies through GLY, causing a concentration-dependent reduction in the binding of motor-protein kinesin to microtubules [22, 32]. Consistent with this hypothesis, there are studies indicating that a stable metabolite may be involved in ACR toxicity on germ cells, rather than ACR itself. For example, the long-term administration of ACR induces chromatid exchanges and breaks in spermatogonia of mice, whereas adverse effects are not remarkable during short-term administration [33]. It has also been reported that reproductive toxicity is less severe when a single high dose of ACR is administered compared to recurrent low doses [34]. In addition, ALKarim *et al*. [35] determined that long-term exposure to a 60 μg/kg/day dose of ACR was associated with cystic ovarian changes and degenerative changes of the zona pellucida, granulosa cells and oocytes in post-weaning Sprague Dawley rats. In light of these data, we conclude that short-term or single-dose exposures to ACR are not solely responsible for toxicity. Rather, one of the major metabolites of ACR causes this effect.

To test whether GLY is involved in ACR toxicity on female germ cells, isolated GV-stage oocytes were treated directly with 25 or 250 μM GLY for 18 hours *in vitro*. GLY exposure resulted in the degeneration of all oocytes, indicating that ACR toxicity may occur through GLY metabolism. It is known that the conversion of ACR to GLY is catalyzed by the CYP2E1 enzyme, a member of cytochrome P-450 family [34]. One of the essential questions of this study is where this conversion occurs. As indicated above, ACR blocks the *in vitro* oocyte maturation process in cumulus-enclosed oocytes but not in denuded oocytes. Therefore, the existence of the CYP2E1 enzyme in either granulosa cells or oocytes appears to be important. According to a study by Sobinoff *et al*. [36], granulosa cells and oocytes from control C57BL/6 mice do not have CYP2E1 immunopositivity, whereas oocyte nuclei show a strong CYP2E1 positivity following smoke exposure. Therefore, we hypothesize that CYP2E1 enzyme-mediated ACR-to-GLY conversion may be an inducible situation and that cumulus cells may induce this conversion. In a study by Ghanayem *et al*. [34], CYP2E1-null and wild-type male mice were treated with 0, 12.5, 25 and 50 mg/kg/day ACR for 5 days and then mated to untreated females. ACR administration to CYP2E1-null mice resulted in healthy offspring [34], indicating that ACR itself is not toxic to the male reproductive system in tested doses, but rather its metabolite GLY is. Ghanayem *et al*. [34] also reported that the number of pregnant females and live fetuses decreased in females mated to wild-type mice that can metabolize ACR to GLY. The results, indicating ACR-GLY conversion, of our *in vivo* and *in vitro* ACR experiments are consistent with the findings of Ghanayem *et al*. [34] Although the results of a study by Liu. *et al*. [26] conflict with this hypothesis, the presence of granulosa cells in culture conditions and the CYP2E1 enzyme activity of granulosa cells should be considered when *in vitro* ACR results are compared to our study, in which only denuded oocytes were studied.

In conclusion, since the direct exposure to ACR does not have toxic effects on mouse oocytes whereas systemic exposure does, we hypothesize that ACR toxicity is caused by its metabolite GLY and we believe that future studies focusing on ACR metabolites, particularly focusing on microtubule polymerization dynamics and microtubule related proteins, should be performed. In one study, internal doses of ACR and GLY were evaluated in humans. Tolerable daily intake (TDI) for neurotoxicity for ACR was estimated to be 40 μg/kg-day whereas the TDI for cancer was estimated to be 2.6 and 16 μg/kg/day for ACR and GLY levels, respectively [37]. A dose–response curve has not been assessed for ACR or GLY in humans, in terms of female fertility. In view of our results, we strongly believe that chronic exposure to ACR must be taken into consideration and a TDI for reproductive toxicity should be estimated for both ACR and GLY in future studies. We also believe that since ACR has tissue-specific genotoxic effects, these effects should be investigated in the ovary, particularly in folliculogenesis. We have initiated a prospective study aiming to measure acrylamide and/or glycidamide levels in the follicular fluid from couples undergoing in vitro fertilization treatment in terms of the effects of these chemicals on female infertility.
